# Ultra-processed foods consumption, depression, and the risk of diabetes complications in the CARTaGENE project: a prospective cohort study in Quebec, Canada

**DOI:** 10.3389/fendo.2023.1273433

**Published:** 2024-01-09

**Authors:** Akankasha Sen, Anne-Sophie Brazeau, Sonya Deschênes, Hugo Ramiro Melgar-Quiñonez, Norbert Schmitz

**Affiliations:** ^1^ School of Human Nutrition, McGill University, Sainte-Anne-de-Bellevue, QC, Canada; ^2^ Douglas Research Centre, Douglas Mental Health University Institute, Montreal, QC, Canada; ^3^ University College Dublin (UCD) School of Psychology, University College Dublin, Dublin, Ireland; ^4^ Department of Psychiatry, McGill University, Montreal, QC, Canada; ^5^ Department of Population-Based Medicine, Tuebingen University, Tuebingen, Germany

**Keywords:** ultra-processed foods, depressive symptoms, type 2 diabetes complications, interaction, CARTaGENE

## Abstract

**Introduction:**

This study aimed to assess the association between depression, ultra-processed food consumption (UPFs), and the risk of developing diabetes-specific complications in adults with type 2 diabetes (T2D).

**Methods:**

Baseline data came from the CARTaGENE study, a health survey of adults (40–69 years) in Quebec, Canada. The incidence of T2D complications was examined in N= 683 participants with T2D without complications at baseline by linking survey data with administrative health data. Food and drink consumption was assessed using the Canadian Diet History Questionnaire and categorized by NOVA classification. Participants were categorized into tertiles of UPFs consumption. Depression was defined as having elevated depressive symptoms based on the Patient Health Questionnaire-9 or the use of antidepressant medications. Cox regression models were used to estimate the associations between UPFs, depression, and T2D complications.

**Results:**

In total, 105 individuals developed diabetes-related complications over a 7-year period. Participants with high depressive symptoms and high UPFs consumption had the highest risk for diabetes complications (adjusted hazard ratio (aHR) 2.07, 95% CI: 0.91 – 4.70), compared to participants with low depressive symptoms and low UPFs consumption. Higher risks for diabetes complications were observed when high depressive symptoms and antidepressant use were combined with high UPFs consumption (aHR 2.59, 95% CI: 1.32 – 5.06).

**Conclusion:**

This study indicates that those with co-occurring depression and high UPFs consumption have a greater risk of diabetes complications. Early management and monitoring of both risk factors might be essential to prevent diabetes complications.

## 1 Introduction

Type 2 diabetes (T2D) is a chronic metabolic condition which requires intensive self-care management (1). Adopting and/or maintaining a healthy diet remains one of the main strategies for the management of T2D and its complications (2). Research has demonstrated that following healthy diets such as the Mediterranean diet (high in olive oil, fruit, nuts, vegetables, and cereals intakes) can reduce the risk of micro-and macrovascular complications among individuals with T2D (3–6).

Recently, in many modern food systems, there has been a nutritional transition characterized by an increase in the consumption of ultra-processed foods (UPFs) as a replacement for fresh foods (7). UPFs are defined as “multi-ingredient industrial formulations” which are characterized by low nutritional quality, high energy density, high saturated and trans fats content, added sugars and salt, and low protein, dietary fiber, and micronutrients (7, 8). Further, UPFs are often designed in a way to encourage eating them in combination (e.g., savory snacks with soft drinks), which can result in excessive caloric intake (7, 8). It has been reported that in higher-income countries, such as the United States and Canada, UPFs can contribute to half of the daily dietary energy intake (9, 10). Higher consumption of UPFs can increase the risk of numerous chronic conditions such as T2D, metabolic syndrome, depression, all-cause mortality, and cardiovascular diseases (9).

Among individuals with T2D, UPFs consumption may increase the risk of developing complications related to T2D. For instance, a recent study found that in individuals with T2D, high consumption of processed foods was associated with poor glycemic control and a greater likelihood of microvascular complications (11). UPFs are associated with elevated levels of glucose (12), which can result in the development of advanced glycation end-products (AGEs). AGEs can activate inflammatory signaling cascades and, consequently, have a crucial role in the pathogenesis of diabetes complications (13).

T2D is a multifactorial disease with psychological complications in addition to physical complications. The risk of developing depressive symptoms is more common in individuals with T2D than in the general population (14). Comorbid depression among individuals with T2D is associated with adverse health outcomes such as micro-and macrovascular complications and higher mortality rates (15). A meta-analysis of longitudinal studies showed that depression was linked with an increased risk of microvascular (HR=1.33; 95% CI: 1.25–1.41) and macrovascular complications (HR=1.38; 95% CI: 1.30–1.47) among adults with T2D (15).

Further, persons with comorbid depression and T2D might have more difficulties following a healthy diet, thereby potentially further increasing the risk of complications (11). Prior research has demonstrated that a history of depression and higher severity of depression was associated with higher emotional and uncontrolled eating, often leading to higher calorie consumption (16). A previous study has also reported an association between depression and high UPFs consumption (17).Consumption of unhealthy foods such as UPFs and high depressive symptoms can independently increase the risk of diabetes-related complications among individuals with T2D (9, 15). It is currently unknown whether high depressive symptoms among individuals with T2D compounds the potential impact of UPFs consumption on the risk of diabetes-related complications. It is possible that depressive symptoms and UPFs consumption may exacerbate the physiological processes, such as systemic inflammation which is risk factor for the T2D and its complications (18–20). Moreover, in a previous study, we found an important interaction between depressive symptoms and UPFs consumption on the risk of developing T2D (21). Adults with both depressive symptoms and high UPFs consumption had a higher risk of developing T2D within a seven-year interval than those without depressive symptoms and with low UPFs consumption (21).

As a next step, we aim to investigate a potential additive interaction between UPFs consumption and depressive symptoms on the incidence of diabetes-related complications in adults with T2D. The combination of depression and consumption of UPFs might not only increase the risk of developing T2D but might also increase the risk of developing diabetes-specific complications in adults with T2D. We, therefore, hypothesized that individuals with T2D with both depressive symptoms and high UPFs consumption at baseline would have a higher risk of developing micro-and macrovascular complications, compared to those without depressive symptoms and with low UPF consumption.

## 2 Methods

### 2.1 Study population

The sample was drawn from the baseline CARTaGENE (CaG) (2009–2010) study (22). CaG is a community health survey that gathered detailed information on health, lifestyle, and sociodemographic information, physiological measures, and biological samples from urban areas of Quebec, Canada (22). Participants aged 40–69 years at baseline were randomly recruited from the Régie de l’assurance maladie du Québec (RAMQ), a governmental health insurance database in the Canadian province of Quebec that provides universal health insurance for residents. Details of the study, such as recruitment, enrollment, and data collection methods, are described elsewhere (22). Briefly, the CaG survey design defined by two age groups, gender, and forward sortation area (defined by 3-digit postal codes). Probability proportional to size was used to describe quotas for each stratum. Participants were excluded if they were not registered in the RAMQ database, those residing outside selected regions, individuals in First Nations Reserves or long-term health care facilities or were in prison (22). Various strategies were employed to ensure response rates and minimize attrition, such as (i) utilizing the reputable governmental body RAMQ to handle participant contact and identifying information, (ii) implementing systematic methods for contact, scheduling, and reminders, and (iii) offering a financial compensation of $45 (22). The recruitment process involved a call center at RAMQ to prevent the transfer of identifying information to CaG. Information packages were initially mailed, followed by telephone contact to enroll participants and schedule clinical assessment site interviews. A total of 20, 007 participants provided informed consent to participate in the CaG cohort study and agreed to link their data with the RAMQ database (22). Ethical approval was provided by the Douglas Mental Health University Institute Research Ethics Board and the St. Justine Hospital Research Ethics Board. Follow-up data referring to T2D complication incidence were obtained by linking participants with diagnostic codes from the RAMQ database.

### 2.2 Depressive symptoms

Depressive symptoms experienced within the past two weeks were measured using the Patient Health Questionnaire-9 (PHQ-9) (23). The PHQ-9 consists of nine questions related to vegetative, emotional, behavioral, and cognitive symptoms of depression. Responses are rated on a 4-point scale ranging from 0 “not at all” to 3 “every day”, with a summary score ranging from 0 to 27, with higher scores reflecting greater depressive symptom severity. The PHQ-9 has shown good agreement with a clinical diagnosis of major depressive disorder and good validity and reliability (23). In the present study, elevated depressive symptoms were defined as having a PHQ-9 summary score of 6 and higher, which includes mild to severe depressive symptoms. This cut-off score has been used in previous studies included in the CaG cohort (24, 25). When compared with the fully structured interviews for major depressive disorder, a PHQ-9 cut-off of 6 has a sensitivity of 0.91 and specificity of 0.61 (26).

### 2.3 Antidepressant use

Participants brought their current medication or reported their current medication at the baseline CaG interview. Medication was classified as an antidepressant based on the medication name (27).

### 2.4 Dietary intake assessment

Dietary intake in the CaG survey was assessed at baseline using the Canadian-adapted diet history questionnaire II (C-DHQ II) (22). C-DHQ II is a validated food frequency questionnaire (FFQ) which reflects food availability, brand names, nutrition composition, and food fortification in Canada (28, 29).

Frequency of consumption and portion sizes are defined for most of the food items in FFQ. Daily consumption of each FFQ food item was computed based on one of four units of time, depending on which answer choice was selected: year, month, week, or day (30). To calculate the daily consumption of each FFQ item, consumption frequency of the items was first converted into daily equivalents such never = 0; 1-6 times per year = 0.01; 7-11 times per year = 0.02; 1 time per month = 0.03; 2-3 times per month = 0.07; 1 time per week = 0.14; 2 times per week = 0.29; 3-4 times per week = 0.48; 5-6 times per week = 0.74; 1 time per day = 1; 2 or more times per day = 3, as specified by the C-DHQII database (30). Secondly, portions of consumed food items were converted into grams by using the nutrient database for the C-DHQII (30). Portions are sex-specific and based on the percentiles of intake reported in the Canadian Community Health Survey (CCHS) – Cycle 2.2 Nutrition (28, 29). The consumed amount for every food item was then calculated by multiplying the frequency per day and grams of consumption. In the present analysis, food items without portion size and items such as vitamins, minerals, or herbal supplements were excluded. Further, items of the C-DHQ II with missing information were filled in with zero imputation, based on the assumption that non-response to the items may be because those items were not consumed by the participants (31).

Every C-DHQ II reported food and beverage item was categorized into one of the four NOVA classification groups. NOVA is not an acronym, but a classification system that groups foods according to the nature, extent, and purpose of the industrial processing (7). Foods were classified into four different groups: 1) unprocessed or minimally processed foods which includes fruit and vegetables, grains (cereals), fresh or pasteurized milk products, seeds without oil and salt, legumes, meat, and fish; 2) processed culinary ingredients such as salt, sugar, vegetable oil, and butter; 3) processed foods, such as canned vegetables and fruits, cheeses, and freshly made bread; and 4) ultra-processed foods and drinks (UPFs) that were prepared mostly or entirely from substances derived from foods, derived from food constituents, or produced in the laboratories from food substrates or other organic sources. Examples of products are ready-to-eat meals, carbonated drinks, biscuits, processed meat, and sugared milk and fruit drinks (7).

To estimate the frequency of consumption of UPFs (grams/day), we summed the amount consumed (grams/day) of each food and beverage item classified in the fourth category of the NOVA classification (a total of 30 foods and seven beverage items). Next, we divided the sample into tertiles according to the total consumption of UPFs (grams/day). Low and middle tertiles were merged as one group for analysis (21).

### 2.5 Incidence of T2DM complications

The study outcomes included micro-and macrovascular diabetes complications. Complications were assessed using diagnostic codes in the RAMQ billing database. Diagnostic codes were based on the World Health Organization’s International Classification of Diseases, 9^th^ or 10^th^ edition (ICD-9 and ICD-10, respectively). Codes for micro-and macrovascular diabetes complications in ICD-9 and ICD-10 were based on prior literature and can be found in **Supplementary Table 1**. For the main analysis, micro-and macrovascular complications were combined. Participants were followed for up to seven years using administrative data from the date of their CARTaGENE baseline assessment. The date of the first diagnosis for micro-and macrovascular diabetes complications was recorded. Observational time was calculated from the day of baseline assessment to the day of complication onset, the date of death, or the study end date of December 31, 2016.

### 2.6 Confounders

Potential confounders include sociodemographic characteristics (age, sex, annual household income, education, and self-reported ethnicity (white was compared with others groups for analysis), behavioral factors including alcohol consumption, defined as whether participants consume alcohol daily or not, smoking (“currently smokes daily or occasionally”, “past smoker”, or has “never smoked”), physical activity (five or more-day moderate activity in a week or three or more vigorous in a week), and body mass index (BMI, continuous) (15).

### 2.7 Statistical analysis

#### 2.7.1 Inclusion criteria

**Figure 1:**
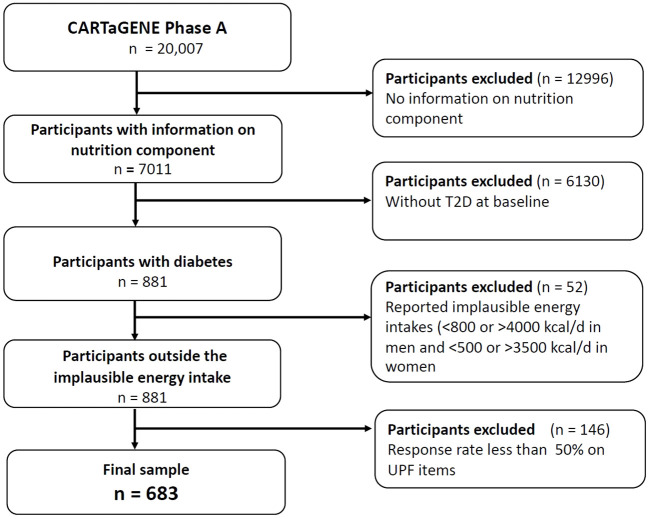
Flow diagram of the final sample for the analysis.

Only CaG participants with information on the nutrition component, depressive symptoms and diabetes status at baseline were included (n = 7,011) (21). Furthermore, the sample was restricted to participants with diabetes and without diabetes complications at baseline (n = 881). Diabetes was self-reported based on a diagnosis made by a physician on a positive response to the following question: ‘Has a doctor ever told you that you had diabetes?’ or HbA1c levels equal to or above 6.5 during the CAG baseline assessment. We excluded all participants who reported implausible energy intakes <800 or >4000 kcal/d in men and <500 or >3500 kcal/d in women (n = 52) as reported in previous research (32). Implausible reporting, particularly underreporting, is a commonly recognized limitation of dietary assessment methods; participants tend to underestimate their total energy intakes and underreport intakes of foods that are deemed unhealthy or socially undesirable, such as foods that are high in fat and refined carbohydrates (32). Further, we excluded participants whose response rates were less than 50% on the UPFs items (n = 146). A total of N = 683 participants were included for the analyses (**Figure 1**). Moreover, we performed two sensitivity analyses, first with a 40% response rate on UPFs items (sample size n = 814) and second with a 60% response rate on the UPFs items (sample size n = 561) to test the robustness of the study.

Cox proportional hazards models were conducted to examine the univariate associations between UPFs consumption, depressive symptoms, and antidepressant use with diabetes complications incidence. Micro-and macrovascular complications were combined for the analysis due to small sample size. However, they were also examined separately in secondary analysis.

To evaluate the potential additive interaction on the incidence of diabetes complications, four groups were defined based on the presence/absence of depressive symptoms and low/high intake of UPFs. The groups were: 1) lower/middle tertile of UPFs consumption and low depressive symptoms (LUND as the reference group), 2) lower/middle tertile of UPFs consumption and elevated depressive symptoms (LUD), 3) higher tertile of UPFs consumption and low depressive symptoms (HUND), and 4) higher tertile of UPFs consumption and elevated depressive symptoms (HUD).

Further, an additional analysis was performed combining depressive symptoms with antidepressant medications as an indicator for depression. Similarly to our primary analyses, four groups were created: 1) lower/middle tertile of UPFs consumption and low depressive symptoms and no antidepressant use (LUNDA as the reference group), 2) lower/middle tertile of UPFs consumption and elevated depressive symptoms or antidepressant use (LUDA), 3) higher tertile of UPFs consumption and low depressive symptoms and no antidepressant use (HUNDA), and 4) higher tertile of UPFs consumption and elevated depressive symptoms or antidepressant use (HUDA). All Cox regression analyses were performed in unadjusted models, in models adjusted for age and sex only, and in fully adjusted models for all the confounders described above. Hazard ratios [HRs] with 95% confidence intervals are reported. Missing information on the covariates was imputed using the fully conditional specification with discriminant or logistic methods using PROC MI procedure in SAS. Cox regression analyses were conducted using SPSS software.

## 3 Results

The main food group contributors to UPFs intake are shown in **Table 1**. Overall, mean (SD) consumption of the UPFs was 276.9 (SD 421.0) g/d, and mean consumption in lower, middle, and highest tertiles was 71.5 (2 SD 3.6) g/d, 154.2 (SD 29.8) g/d, and 604.0 (SD 605.3) g/d, respectively.

**Table 1:** Contribution of each food group to the total amount of ultra-processed foods consumed in the CARTaGENE study cohort (*n=683*).

Food groups (n= 37)	Contribution to total ultra-processed foods intake (%) *	Daily amount consumed mean g/d (SD)
Beverages (n=7)		
Dairy beverages	4.2	11.6 (38.8)
Soft/isotonic drinks	51.2	141.8 (384.3)
Fruit drinks	3.1	8.5 (54.4)
Solid Foods (n = 30)		
Processed meat	4.8	13.3 (30.9)
Fast food and ready to eat	10.8	29.9 (32.9)
Breakfast cereals	3.7	10.3 (16.2)
Cookies, biscuits, muffins, and cake	10.4	28.7 (40.0)
Potato chips and salty snacks	3.0	8.2 (9.7)
Confectionery and chocolate	2.0	5.4 (9.5)
Ketchup, salad dressing and similar	3.9	10.7 (13.5)
Ice-cream	1.9	5.3 (9.8)
Jelly and jams products	1.2	3.2 (6.6)
Total	100	276.9 (421.0)

*Contribution (%) of each food group/beverage to the total consumption of ultra-processed food was calculated by dividing the amount (g/d) of each food group by the total amount of ultra-processed foods (g/d) multiplied by 100.

**Table 2** displays the characteristics of the sample. The baseline data reveals a mean age of 55.5 years (SD = 7.5), with 52.6% being female and 93.3% identifying as white. A total of 105 (15.4%) individuals developed diabetes-related complications during the observation period. Using the categorical classifications for groups based on UPFs and PHQ-9 scores, there were 395 (57.0%) participants in LUND group (reference group); 60 (8.9%) participants in LUD group; 191 (28.8%) participants in HUND group; and 37 (5.3%) participants in HUD group. Participants in the HUD group exhibited a higher percentage of lower-income levels and a lower percentage of postsecondary education compared to the other group. Additionally, individuals in the HUD group were more likely to be daily or occasional smokers and physically inactive compared to the other group. Moreover, the HUD group had a higher mean intake of UPFs 615.2 (478.2) g/d, and a higher BMI 31.0 (6.2) as compared to the other groups.

**Table 2:** Baseline characteristics of the study sample.

	LUND (n = 395)	LUD (n = 60)	HUND (n = 191)	HUD (n = 37)	Total (n = 683)
Age, mean (SD)	56.2 (7.5)	55.2 (7.5)	54.5 (7.5)	53.7 (5.9)	55.5 (7.5)
Sex n (%)					
Male	163 (41.3)	18 (30.0)	122 (63.9)	21 (56.8)	324 (47.4)
Female	232 (58.7)	42 (70.0)	69 (36.1)	16 (43.2)	359 (52.6)
Household income n (%)					
Lower income level (<49,999 $)	123 (31.1)	23 (38.3)	55 (28.9)	20 (54.1)	221 (32.4)
Middle income level (50,000 – 149,999 $)	231 (58.5)	33 (55.0)	117 (61.9)	15 (40.5)	396 (58.0)
High income level (>150,000 $)	41 (10.4)	4 (6.7)	19 (9.9)	2 (5.4)	66 (9.7)
Postsecondary education n (%)					
No	89 (22.5)	19 (31.7)	51 (26.7)	15 (40.5)	174 (25.5)
Yes	306 (77.5)	41 (68.3)	140 (73.3)	22 (59.5)	509 (74.5)
Born in Canada n (%)					
No	43 (10.9)	14 (23.3)	11 (5.8)	2 (5.4)	70 (10.2)
Yes	352 (89.1)	46 (76.7)	180 (94.2)	35 (94.6)	613 (89.8)
Ethnicity n (%)					
Other	25 (6.3)	10 (16.7)	6 (3.1)	5 (10.9)	60 (6.7)
White	370 (93.7)	50 (83.3)	185 (96.9)	32 (86.5)	637 (93.3)
Marital status n (%)					
Married/partner	270 (68.4)	38 (63.3)	128 (67.0)	26 (70.3)	462 (67.6)
Single	53 (13.4)	9 (15.0)	31 (16.2)	7 (18.9)	100 (14.6)
Divorced/separated/widowed	72 (18.5)	13 (21.7)	32 (16.8)	4 (10.8)	121 (17.7)
Daily alcohol consumption n (%)					
No	342 (86.6)	55 (91.7)	179 (93.7)	35 (94.6)	611 (89.5)
Yes	53 (13.4)	5 (8.3)	12 (6.3)	2 (5.4)	72 (10.5)
Smoking status n (%)					
Daily and occasional	45 (11.4)	11 (18.3)	38 (19.9)	8 (21.6)	102 (14.9)
Past smoking	184 (46.6)	19 (31.7)	82 (42.9)	16 (43.2)	301 (44.1)
Never smoking	166 (42.0)	30 (50.0)	71 (37.2)	13 (35.1)	280 (41.0)
Physical activity n (%)					
Yes	152 (38.5)	17 (28.3)	83 (43.5)	9 (24.3)	261 (38.2)
No	243 (61.5)	43 (71.7)	108 (56.5)	28 (75.7)	422 (61.8)
UPF consumption grams/day, mean (SD)	112.0 (49.7)	118.7 (46.8)	601.8 (628.0)	615.2 (478.2)	276.9 (421.0)
BMI, mean (SD)	27.8 (5.4)	29.0 (6.6)	29.8 (5.9)	31.0 (6.2)	28.6 (5.9)
Diabetes complication n (%)	52 (13.2)	10 (16.7)	34 (17.8)	9 (24.3)	105 (15.4)

Results reported as mean ± SD for continuous data and n (%) for categorical data.

LUND, lower/middle tertile of ultra-processed foods consumption and low depressive symptoms; LUD, lower/middle tertile of ultra-processed foods consumption and high depressive symptoms; HUND, higher tertile of ultra-processed foods consumption and low depressive symptoms; HUD, higher tertile of ultra-processed foods consumption and high depressive symptoms; UPFs, Ultra-processed foods.

**Table 3** describes the results of three univariate Cox regression analyses examining UPFs, depressive symptoms, and antidepressant use. Participants in the highest tertile of UPFs consumption had the greatest hazard ratios for developing complications in the fully adjusted model (HR=1.56, 95% CI: 0.92-2.62); however, the CI were overlapping with the one. Similarly, the CI overlapped with one in a fully adjusted model for depressive symptoms (PHQ-9>= 6) and for antidepressant use with HRs of 1.45 (95% CI: 0.84- 2.51) and 1.61 (95% CI: 0.86 – 3.00) respectively.

**Table 3:** Results of Cox regression for UPFs consumption and depression assessed using PHQ9 and antidepressant for incident T2D complications.

Groups	N	Unadjusted Model, HR (95% CI)	Age- and Sex-Adjusted Model, HR (95% CI)	Fully Adjusted Model, HR (95% CI) *
Model 1: UPFs consumption univariate association				
Lower tertile of UPFs consumption	227	Reference	Reference	Reference
Middle tertile of UPFs consumption	228	0.99 (0.60 -1.63)	1.08 (0.65 -1.79)	1.15 (0.69 – 1.93)
Higher tertile of UPFs consumption	228	1.32 (0.83– 2.21)	1.54 (0.95 -2.50)	1.56 (0.92 - 2.62)
Model 2: Depression univariate association				
PHQ-9 summary score (< 6) Low	586	Reference	Reference	Reference
PHQ-9 summary score (>= 6) High	97	1.57 (0.95 -2.59)	1.63 (0.98 – 2.71)	1.45 (0.84 – 2.51)
Model 3: Antidepressant use univariate association				
Anti-depressant use NO	625	Reference	Reference	Reference
Antidepressant use YES	58	1.54 (0.86- 2.78)	1.57 (0.87 – 2.81)	1.61 (0.86 – 3.00)

UPFs, Ultra-processed foods; PHQ-9, Patient Health Questionnaire-9.

*Fully adjusted model is adjusted for the following variables: age, sex, household income, education, ethnicity, born in Canada, smoking status, physical activity, daily alcohol consumption and BMI.

**Table 4** shows results obtained from the additive interaction analysis, with the reference category in model 1 set as the LUND group. In HUD group, 24.3% of individuals developed complications. In the age and sex-adjusted model, the HUD group had a 2.4-fold increased risk of developing complications as compared to the LUD and HUND group. However, in the fully adjusted model, HUD group HR was 2.07 (95% CI: 0.91 – 5.06), and CI overlapped with one.

**Table 4:** Results of Cox regression for UPFs consumption and depression assessed using PHQ9 and antidepressant joint association for incident T2D complications.

Model 1 UPFs consumption lower & middle tertile combined and depressive symptoms joint association					
Groups	N	Incident complications (N)	Unadjusted	Age- and Sex-Adjusted Model, HR (95% CI)	Fully Adjusted Model, HR (95% CI)
LUND	395	52	Reference	Reference	Reference
LUD	60	10	1.49 (0.75- 2.94)	1.48 (0.75 – 2.94)	1.39 (0.69- 2.80)
HUND	191	34	1.29 (0.83 -2.00)	1.40 (0.90 – 2.20)	1.41 (0.88 - 2.25)
HUD	37	9	2.07 (1.02 – 4.20)	2.43 (1.18 – 4.99)	2.07 (0.91 – 4.70)
Model 2 UPFs consumption lower & middle tertile combined and depressive symptoms/Antidepressant use joint association					
LUNDA	367	49	Reference	Reference	Reference
LUDA	88	13	1.30 (0.70 - 2.42)	1.29 (0.70 - 2.40)	1.30 (0.69 – 2.45)
HUNDA	179	29	1.16 (0.73 -1.84)	1.25 (0.78 - 2.01	1.27 (0.78 – 2.09)
HUDA	49	14	2.37 (1.30 – 4.30)	2.82 (1.53 – 5.18)	2.59 (1.32 – 5.06)

*Fully adjusted model is adjusted for the following variables: age, sex, household income, education, ethnicity, born in Canada, smoking status, physical activity, daily alcohol consumption and BMI.

LUND, lower/middle tertile of ultra-processed foods consumption and low depressive symptoms; LUD, lower/middle tertile of ultra-processed foods consumption and high depressive symptoms; HUND, higher tertile of ultra-processed foods consumption and low depressive symptoms; HUD, higher tertile of ultra-processed foods consumption and high depressive symptoms; LUNDA, lower and middle tertile of ultra-processed foods consumption and low depressive symptoms and no antidepressant use; LUDA, lower and middle tertile of ultra-processed foods consumption and high depressive symptoms or antidepressant use; HUNDA, higher tertile of ultra-processed foods consumption and low depressive symptoms and no antidepressant; HUDA, higher tertile of ultra-processed foods consumption and high depressive symptoms or antidepressant.

Further in model 2, when elevated depressive symptoms and antidepressant medication were combined as indicators for depression, 28.6% of individuals developed T2D complications. And similarly greater risk for T2D complications was found in the HUDA group in the model adjusted for age and sex (2.82, 95% CI: 1.53-5.18). Moreover, in a fully adjusted model, the HR was 2.59 (95% CI: 1.32-5.06).

We also performed separate analyses for microvascular complications. The results are not presented in the tables because of the small sample size. For micro complications, there were 37 individuals in group HUD, and out of these individuals, only 8 individuals developed the micro complication with an adjusted HR of 2.64 (95% CI: 1.06 – 6.54) (**Supplementary Table 2**).

Moreover, two sensitivity analysis showed similar results, suggesting that participants in the depressive symptoms and UPFs consumption groups had higher hazard ratios for developing diabetes complications than those with either condition alone (**Supplementary Tables 3, 4**).

## 4 Discussion

In this prospective study, we examined the associations between UPFs consumption, depressive symptoms, and the risk of developing T2D complications among middle-aged adults by linking survey data with administrative data. We found that individuals with depressive symptoms and higher consumption of UPFs at baseline had a higher risk of developing T2D related micro-and macro complications in a model adjusted for sex and age as compared to those with neither condition, and this risk estimate was higher than those with depressive symptoms only and those with high UPF consumption only. Further, when depressive symptoms and higher consumption of the UPFs group were controlled for additional confounders in the fully adjusted model, the HRs were lowered and included 1.00 in the CI. However, when depressive symptoms and antidepressant medication use were combined as indicators for depression, then the combination of both resulted in the CI that did not include 1.00 in the fully adjusted model. These results suggest an interaction between depression and UPFs consumption in relation to an increased risk of diabetes-related complications.

To our knowledge no study in the past directly investigated this interaction. One study has reported that T2D individuals with food addiction, which is associated with UPFs consumption (33), had a greater prevalence of diabetes retinopathy, neuropathy, nephropathy, and depressive symptoms compared to those without food addictions (11).

There are several pathways in which depression or depressive symptoms may be associated with an increased risk of developing diabetes complications. One of the potential pathways by which depression among T2D individuals might increase the risk of diabetes-related complications is through suboptimal diabetes management (14, 15). It has been reported that individuals with T2D and high depressive symptoms tend to have lower adherence to medication, diet, and exercise than individuals with T2D alone (15). In addition, depression can be accompanied by behavioral changes, such as reduced self-care and medication adherence, increased intake of high-calorie food, smoking, reduced physical activity, and increased sedentary behaviors (15). These behaviors might have more detrimental effects in the context of diabetes, possibly resulting in poor glycemic control, which, in turn, may be associated with an increased risk of complications (15).

Diabetes with comorbid depressive symptoms is associated with increased hypothalamic–pituitary–adrenal axis and sympathetic nervous system activation (14). Further, increased insulin resistance and high concentration of inflammatory markers may lead to complications in individuals with comorbid diabetes and depression (14, 18). Depressive symptoms and UPFs are also independently associated with inflammatory markers such as C-reactive protein, tumor necrosis factor-α, interleukin-1, and interleukin-6 levels (18, 19). UPFs often occur within high obesogenic environments and have higher glycemic loads (18, 19). These diets may induce hyperglycemia, which is associated with increased pro-inflammatory cytokines, including IL-6 and TNF-α, leading to insulin resistance by disruptions in insulin signalling and subsequently might increase the risk of the diabetes complications (13). Besides the nutritional aspects of UPFs, recent concern has emerged on changes in microbiota induced by non-nutritive components, mainly by flavors, emulsifiers, and thickeners, which may provoke gut dysbiosis and initiate inflammation in the gut (34). However, more research is needed to better understand the relative effects of UPFs on diabetes related complication incidence.

Furthermore, antidepressants use is one of the standard treatments for depressive disorders (35). However, certain antidepressants can increase the risk of body weight and poor glycemic control (35), which might lead to diabetes-related complications (36). Our study shows that; when antidepressant use and depressive symptoms were combined with high UPFs consumption, the risk of diabetes complications was higher than the depressive symptoms combined with high UPFs consumption.

### 4.1 Strengths and limitation

Strengths of this study include its prospective design, the use of two different measures for depression, the combined use of survey data with administrative health data, and adjustment for potential confounders. Further, two sensitivity analyses using two different response rates on UPFs consumption were conducted to assess the robustness of the study findings. Acknowledging that the data is 13 years old, we also acknowledge the general challenge of low response rates to food frequency questionnaires in epidemiological studies focusing on nutrition and health outcomes. Despite the age of the data, this study plays a crucial role in addressing a gap in the literature. By examining the combined impact of depression and UPF consumption, two significant modifiable risk factors, it provides valuable insights into how they jointly influence the risk of diabetes related complications.

There are also various limitations that should be noted. First, the C-DHQ II used in this study was designed to evaluate the intake of major food groups, energy, and macronutrients, not specifically to collect data about the NOVA classification of UPFs consumption. Further, there is also limitation related to NOVA classification. Because of its complex and multidimensional definition of levels of food processing, there is a potential for introducing ambiguity and variations in interpretation related to UPF (37). Assessment of the diet intake was self-reported and only measured at the baseline; therefore, it might be possible that participants change their intake of ultra-processed foods during the follow-up. Participants of the CaG study were volunteers in a nutrition component, and thus it may be possible that these individuals were more interested in nutritional issues and healthy lifestyles than the general population. And it might be possible that their consumption of UPFs may be lower compared to the general population, which may underestimate the risk investigated in our study. Depressive symptoms were assessed at baseline only. The PHQ-9 is a self-report scale that measures symptoms of depression experienced in the past two weeks and does not consider the history and treatment of depression. Given that depressive symptoms were not measured during the follow-up, symptoms may vary and change over time. Further, another important limitation is that our analysis does not eliminate the possibility that part of this association stems from a shared pathophysiological factor — specifically, the impact of UPF consumption on both diabetes progression/complications and the onset of depression (9). Moreover, there is also limitation with administrative data. In Canada administrative hospital data are produced by health professionals who review, abstract, and code information from inpatient charts following hospital discharge. One of issue with the administrative data is the undercoding of diabetes and its related complications by physicians which can lead to an incomplete representation of the true prevalence (38).

The individual group sizes were small, and therefore studies with large sample size are needed to replicate the findings. CaG participants were mostly white participants (93.3%) and metropolitan; as a result, generalization of our findings should be made with caution.

### 4.2 Conclusion

To conclude, our study suggests that individuals with co-occurring depression and high UPF consumption may represent a group at risk of developing T2D complications. Thus, this group possibly be benefit from greater monitoring and preventive care. However, future research is needed to disentangle the mechanisms linking depression and UPF consumption to T2D complications. In addition, further research is required to replicate these findings in large samples with longer follow-up periods.

## Data availability statement

Publicly available datasets were analyzed in this study. Data can be obtained from the Cartagene cohort study.

## Ethics statement

The studies involving humans were approved by Douglas Research Ethics Board, Montreal, QC Canada. The studies were conducted in accordance with the local legislation and institutional requirements. The participants provided their written informed consent to participate in this study.

## Author contributions

NS: Conceptualization, Funding acquisition, Methodology, Project administration, Supervision, Writing – review & editing. AS: Conceptualization, Formal Analysis, Methodology, Writing – original draft. AB: Conceptualization, Methodology, Supervision, Validation, Writing – review & editing. SD: Conceptualization, Investigation, Methodology, Supervision, Writing – review & editing. HR-Q: Conceptualization, Methodology, Supervision, Writing – review & editing.
