# Supplementary material for: Ultra-processed foods consumption, depression, and the risk of diabetes complications in the CARTaGENE project: a prospective cohort study in Quebec, Canada
Source: Front Endocrinol (Lausanne). 2024 Jan 9;14:1273433. doi: 10.3389/fendo.2023.1273433 (PMC10803882; doi:10.3389/fendo.2023.1273433)
Supplement: Supplementary file 1 [file Supplementary_file_1.docx]

Supplementary Material

**Ultra-processed foods consumption, depression, and the risk of diabetes complications in the CARTaGENE project: A prospective cohort study in Quebec, Canada**

**Akankasha Sen^1, 2^, Anne-Sophie Brazeau ^1^, Sonya Deschênes ^3^, Hugo Ramiro Melgar-Quiñonez ^1^, Norbert Schmitz ^2, 4, 5 *^**

^1^ School of Human Nutrition, McGill University, 21,111 Lakeshore Road Ste. Anne de Bellevue, Quebec H9X 3V9, Canada

^2^ Douglas Mental Health University Institute, 6875 Bd LaSalle, Quebec, Canada, H4H 1R3

^3^ UCD School of Psychology, University College Dublin, Stillorgan Rd, Belfield, Dublin 4, Ireland

^4^ Department of Psychiatry, McGill University, 1033 Pine Avenue West Montreal, Quebec, H3A 1A1, Canada

^5^ Department of Population-Based Medicine, Tuebingen University, Hoppe-Seyler-Str. 9, 72076 Tuebingen, Germany

*** Correspondence:**Norbert Schmitz, PhD

E-mail: [Norbert.Schmitz@med.uni-tuebingen.de](mailto:Norbert.Schmitz@med.uni-tuebingen.de)

Phone +49-7071 29-88812

**Supplementary Table 1**: Diabetes complication code

| **Diabetes complications** | ICD-9 Diagnosis | ICD-9 codes ^(1,2,3)^ | ICD-10 Diagnosis | ICD-10 codes ^(4,5,6,7)^ |
| --- | --- | --- | --- | --- |
| **Microvascular** |  |  |  |  |
| **Diabetic retinopathy** | Diabetic ophthalmologic disease | 250.5x |  |  |
|  | Background retinopathy | 362.01 |  |  |
|  | Other retinopathy | 362.1 | Diabetic retinopathy | H0-H4, H50-H53, H55, H57-H59, E103, E113, E143  E113, E143 or H360 |
|  | Retinal edema | 362.83 |  |  |
|  | cystoid macular edema/degeneration (CSME) | 362.53 |  |  |
|  | Proliferative retinopathy | 362.02 |  |  |
|  | Retinal Detachment | 361 |  |  |
|  | Blindness | 369 | Vision loss or blindness on one or two eyes | H54 |
|  | Other retinal disorders | 362.81, 362.82 |  |  |
|  | Vitreous hemorrhage | 379.23 |  |  |
| **Nephropathy** | Diabetic nephropathy | 250.4 | Diabetic nephropathy/ kidney disease | E11.2, E14.2 E11.7, E11.8, E14.7, E14.8, R809, N00-N08, N10-N19, N28.9 |
|  | Acute glomerulonephritis | 580 |  |  |
|  | Nephrotic syndrome | 581 |  |  |
|  | Chronic glomerulonephritis | 582 |  |  |
|  | Hypertension, nephrosis | 581.81 |  |  |
|  | Nephritis/nephropathy | 583 |  |  |
|  | Acute renal failure | 584 |  | N17 |
|  | \| Chronic renal failure \| \| --- \| | 585 |  | N18, N19 |
|  | Renal failure NOS | 586 |  |  |
|  | Renal insufficiency | 593.9 |  |  |
|  | Specified diabetic kidney disease | 250.4 | Specified diabetic kidney disease | E10.2, E11.2, E14.2 |
|  | Unspecified diabetic kidney disease | 250.9 | Unspecified diabetic kidney disease | E10.7, E10.8, E11.7, E11.8, E14.7, E14.8 |
| **Neuropathy** |  |  |  |  |
|  | Diabetic neuropathy | 356.9,250.6 | Diabetic neuropathy | E114, E115, E124, E125, E134, E135, E144, E145 |
|  | Amyotrophy | 358.1 |  |  |
|  | \| Cranial nerve palsy \| \| --- \| | 951.0, 951.1, 951.3 |  |  |
|  | Mononeuropathy | 354.0-355.9 |  |  |
|  | \| Charcot’s arthropathy \| \| --- \| | 713.5 |  |  |
|  | Polyneuropathy | 357.2 |  |  |
|  | Neurogenic Bladder | \| 596.54 \| \| --- \| |  |  |
|  | Autonomic neuropathy | 337.0, 337.1 |  |  |
|  | Gastroparesis/diarrhea | 564.5, 536.3 |  |  |
|  | Orthostatic hypotension | 458.0 |  |  |
| **Macrovascular** |  |  |  |  |
| **Cerebrovascular** |  |  |  |  |
|  | TIA, Transient ischemic attack | 435 |  |  |
|  | Stroke | 431, 433, 434, 436 | Stroke | I61, I63–I67 |
| **Cardiovascular** | Atherosclerosis | 440.xx |  |  |
|  | Other IHD (ischemic heart disease) | 411, 415 | Other (ischemic heart disease) | I24 |
|  | Angina pectoris | 413 | Angina pectoris | I20 |
|  | Other chronic IHD (ischemic heart disease) | 414 | Other chronic IHD | I25 |
|  | Myocardial infarction | 410 | Myocardial infarction | I21 |
|  | Ventricular fibrillation, arrest | 427.1, 427.3 | Subsequent myocardial infarction | I22 |
|  | Atrial fibrillation, arrest | 427.4, 427.5 | Certain current complications following acute myocardial infarction | I23 |
|  | Other ASCVD (atherosclerotic cardiovascular disease) | 429.2 |  |  |
|  | Old myocardial infarction | 412 |  |  |
|  | Heart failure | 428 | Heart failure | I46, I50, I11.0, I13.0, I13.2 |
|  | Atherosclerosis, severe | 440.23, 440.24 |  |  |
|  | Aortic aneurysm/dissection | 441 |  |  |
| **Peripheral vascular disease (PVD)** | Diabetic PVD | 250.7 |  | I70.2, I73.1, I73.9, I79.2, E10.5, E11.5, E14.5 |
|  | Other aneurysm, Lower extremity | 442.3 | Chronic arterial occlusion | I70 |
|  | peripheral vascular disease | 443.81, 443.9 |  |  |
|  | Foot wound + complication | 892.1 | Diabetic foot and ulcers | E11.6, E14.4, L00, L03, L08, L97 |
|  | Claudication, intermittent | 443.9 |  |  |
|  | Embolism/thrombosis (LE) | 444.22 |  |  |
|  | Gangrene | 785.4 |  |  |
|  | Gas gangrene | 0.40 |  |  |
|  | Ulcer of lower limbs | 707.1 |  |  |


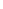

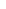

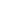

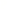

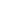

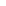


Reference:

1. Young BA, Lin E, Von Korff M, Simon G, Ciechanowski P, Ludman EJ, Everson-Stewart S, Kinder L, Oliver M, Boyko EJ, Katon WJ. Diabetes complications severity index and risk of mortality, hospitalization, and healthcare utilization. The American journal of managed care. 2008 Jan;14(1):15.
2. O'Brien JA, Patrick AR, Caro JJ. Cost of managing complications resulting from type 2 diabetes mellitus in Canada. BMC health services research. 2003 Dec;3(1):1-1.
3. Cheng SW, Wang CY, Ko Y. Costs and length of stay of hospitalizations due to diabetes-related complications. Journal of diabetes research. 2019 Sep 8;2019.
4. Adamsson Eryd S, Svensson AM, Franzén S, Eliasson B, Nilsson PM, Gudbjörnsdottir S. Risk of future microvascular and macrovascular disease in people with Type 1 diabetes of very long duration: a national study with 10‐year follow‐up. Diabetic medicine. 2017 Mar;34(3):411-8.
5. Fukuda H, Mizobe M. Impact of nonadherence on complication risks and healthcare costs in patients newly diagnosed with diabetes. Diabetes research and clinical practice. 2017 Jan 1;123:55-62.
6. Andersson E, Persson S, Hallén N, Ericsson Å, Thielke D, Lindgren P, Carlsson KS, Jendle J. Costs of diabetes complications: hospital-based care and absence from work for 392,200 people with type 2 diabetes and matched control participants in Sweden. Diabetologia. 2020 Dec;63(12):2582-94.
7. Fujihara K, Yamada‐Harada M, Matsubayashi Y, Kitazawa M, Yamamoto M, Yaguchi Y, Seida H, Kodama S, Akazawa K, Sone H. Accuracy of Japanese claims data in identifying diabetes‐related complications. Pharmacoepidemiology and Drug Safety. 2021 May;30(5):594-601.

**Supplementary Table 2:** Results of cox regression for UPFs consumption and depression assessed using PHQ9 and anti-depressant joint association for type 2 diabetes microvascular complication incidence in CARTaGENE

| Groups | N | **Unadjusted** | **Age- and Sex-Adjusted Model, HR (95% CI)** | **Fully Adjusted Model, HR (95% CI) *** |
| --- | --- | --- | --- | --- |
| **Model 1 UPF consumption low/middle tertiles combined and depressive symptoms joint association** | | | | |
| LUND | 295 | Reference | Reference | Reference |
| LUD | 60 | 1.34 (0.56 – 3.20) | 1.34 (0.56 – 3.21) | 1.20 (0.49 – 2.94) |
| HUND | 191 | 1.46 (0.87 – 2.44) | 1.60 (0.94 – 2.72) | 1.57 (0.91 -2.73) |
| HUD | 37 | 2.80 (1.29 – 6.05) | 3.33 (1.52 – 7.29) | 2.64 (1.06 – 6.54) |
| **Model 2 UPF consumption low/middle combined tertile and depressive symptoms/ Antidepressant use joint association** | | | | |
| LUNDA | 362 | Reference | Reference | Reference |
| LUDA | 88 | 1.43 (0.70 – 2.92) | 1.19 (0.54 – 2.60) | 1.16 (0.52 – 2.56) |
| HUNDA | 184 | 1.08 (0.61 – 1.89) | 1.33 (0.75 – 2.35) | 1.32 (0.73 – 2.38) |
| HUDA | 49 | 2.71 (1.37 – 5.37) | 4.06 (2.10 – 7.871) | 3.67 (1.75 – 7.66) |
| LUND, lower/middle tertile of ultra-processed foods consumption and low depressive symptoms; LUD, lower/middle tertile of ultra-processed foods consumption and high depressive symptoms; HUND, higher tertile of ultra-processed foods consumption and low depressive symptoms; HUD, higher tertile of ultra-processed foods consumption and high depressive symptoms; LUNDA, lower and middle tertile of of ultra-processed foods consumption and low depressive symptoms and no antidepressant use; LUDA, lower and middle tertile of ultra-processed foods consumption and high depressive symptoms or antidepressant use; HUNDA, higher tertile of ultra-processed foods consumption and low depressive symptoms and no antidepressant; HUDA, higher tertile of ultra-processed foods consumption and high depressive symptoms or antidepressant.  *Fully adjusted model is adjusted for the following variables: age, sex, household income, education, ethnicity, born in Canada, smoking status, physical activity, daily alcohol consumption and BMI. | | | | |

We performed to two sensitivity analysis, by performing the Cox regressions first with a 40% and second with a 60% response rate on the ultra-processed food and beverage items.

1. 40% response rate 15 questions out of 37 on UPF, sample size n = 814

**Supplementary Table 3 A.**  Results of cox regression for UPFs consumption and depression assessed using PHQ9 and anti-depressant for type 2 diabetes combined complication incidence in CARTaGENE

| Groups | N | **Unadjusted** | **Age- and Sex-Adjusted Model, HR (95% CI)** | **Fully Adjusted Model, HR (95% CI) *** |
| --- | --- | --- | --- | --- |
| **Model 1: UPFs consumption univariate association** | | | | |
| Lower t**ertile o**f UPFs consumption | 271 | Reference | Reference | Reference |
| Middle t**ertile o**f UPFs consumption | 272 | 0.90 (0.57 – 1.40) | 0.93 (0.60 – 1.47) | 0.96 (0.61 – 1.52) |
| Higher t**ertile o**f UPFs consumption | 271 | 1.20 (0.79 – 1.81) | 1.31 (0.85 – 2.02) | 1.28 (0.81 – 2.01) |
| **Model 2: Depression univariate association** | | | | |
| PHQ-9 summary score (< 6) Low | 697 | Reference | Reference | Reference |
| PHQ-9 summary score (>= 6) High | 117 | 1.68 (1.09 – 2.59) | 1.77 (1.15 – 2.75) | 1.63 (1.02 – 2.61) |
| **Model 3: Anti-depressant use univariate association** | | | | |
| Anti-depressant use NO | 745 | Reference | Reference | Reference |
| Anti-depressant use YES | 69 | 1.32 (0.76 – 2.30) | 1.38 (0.79 – 2.42) | 1.38 (0.77 – 2.49) |
| UPFs, Ultra-processed foods; PHQ-9, Patient Health Questionnaire-9.  *Fully adjusted model is adjusted for the following variables: age, sex, household income, education, ethnicity, born in Canada, smoking status, physical activity, daily alcohol consumption and BMI. | | | | |

**Supplementary Table 3 B.** Results of cox regression for UPFs consumption and depression assessed using PHQ9 and anti-depressant joint association for type 2 diabetes combined complication incidence in CARTaGENE

| **Groups** | **N** | **Unadjusted** | **Age- and Sex-Adjusted Model, HR (95% CI)** | **Fully Adjusted Model, HR (95% CI) *** |
| --- | --- | --- | --- | --- |
| **Model 1** **UPFs consumption lower & middle tertile combined and depressive symptoms joint association** | | | | |
| LUND | 472 | Reference | Reference | Reference |
| LUD | 71 | 1.56 (0.87 – 2.79) | 1.60 (0.89 – 2.85) | 1.53 (0.85 – 2.77) |
| HUND | 225 | 1.20 (0.80 – 1.80) | 1.27 (0.84 – 1.92) | 1.25 (0.82 – 1.92) |
| HUD | 46 | 2.14 (1.15 – 3.97) | 2.50 (1.34 – 4.68) | 2.22 (1.08 – 4.54) |
| **Model 2 UPFs consumption lower & middle tertile combined and depressive symptoms/Antidepressant use joint association** | | | | |
| LUNDA | 437 | Reference | Reference | Reference |
| LUDA | 106 | 1.23 (0.72 – 2.11) | 1.27 (0.74 – 2.18) | 1.27 (0.73 – 2.21) |
| HUNDA | 212 | 1.07 (0.70 – 1.64) | 1.24 (0.73 – 1.74) | 1.13 (0.72 – 1.76) |
| HUDA | 59 | 2.29 (1.33 – 3.92) | 2.72 (1.57 – 4.71) | 2.50 (1.36 – 4.58) |
| LUND, lower/middle tertile of ultra-processed foods consumption and low depressive symptoms; LUD, lower/middle tertile of ultra-processed foods consumption and high depressive symptoms; HUND, higher tertile of ultra-processed foods consumption and low depressive symptoms; HUD, higher tertile of ultra-processed foods consumption and high depressive symptoms; LUNDA, lower and middle tertile of of ultra-processed foods consumption and low depressive symptoms and no antidepressant use; LUDA, lower and middle tertile of ultra-processed foods consumption and high depressive symptoms or antidepressant use; HUNDA, higher tertile of ultra-processed foods consumption and low depressive symptoms and no antidepressant; HUDA, higher tertile of ultra-processed foods consumption and high depressive symptoms or antidepressant.  *Fully adjusted model is adjusted for the following variables: age, sex, household income, education, ethnicity, born in Canada, smoking status, physical activity, daily alcohol consumption and BMI. | | | | |

1. 60% response rate 22 questions out of 37 on UPFs, sample size n = 561

**Supplementary Table 4 A.**  Results of Cox Regression for UPFs consumption and Depression Assessed Using PHQ9 and Anti-depressant for type 2 diabetes combined complication incidence in CARTaGENE

| **Groups** | **N** | **Unadjusted** | **Age- and Sex-Adjusted Model, HR (95% CI)** | **Fully Adjusted Model, HR (95% CI) *** |
| --- | --- | --- | --- | --- |
| **Model 1: UPFs consumption univariate association** | | | | |
| Lower t**ertile** Of UPFs consumption | 187 | Reference | Reference | Reference |
| Middle t**ertile** Of UPFs consumption | 187 | 0.94 (0.55 – 1.61) | 1.06 (0.61– 1.81) | 1.10 (0.63 – 1.93) |
| Higher t**ertile** Of UPFs consumption | 187 | 1.35 (0.82 – 2.21) | 1.56 (0.93 – 2.62) | 1.55 (0.89 – 2.71) |
| **Model 2: Depression univariate association** | | | | |
| PHQ-9 summary score (< 6) Low | 697 | Reference | Reference | Reference |
| PHQ-9 summary score (>= 6) High | 117 | 1.48 (0.88 – 2.49) | 1.57 (0.93 – 2.66) | 1.40 (0.80 – 2.47) |
| **Model 3: Antidepressant use univariate association** | | | | |
| Anti-depressant use NO | 745 | Reference | Reference | Reference |
| Anti-depressant use YES | 69 | 1.71 (0.95 – 3.09) | 1.69 (0.93 – 3.05) | 1.70 (0.91 – 3.21) |
| UPFs, Ultra-processed foods; PHQ-9, Patient Health Questionnaire-9.  *Fully adjusted model is adjusted for the following variables: age, sex, household income, education, ethnicity, born in Canada, smoking status, physical activity, daily alcohol consumption and BMI. | | | | |

**Supplementary Table 4 B.** Results of cox regression for UPFs consumption and depression assessed using PHQ9 and anti-depressant joint association for type 2 diabetes combined complication incidence in CARTaGENE

| **Groups** | **N** | **Unadjusted** | **Age- and Sex-Adjusted Model, HR (95% CI)** | **Fully Adjusted Model, HR (95% CI)** |
| --- | --- | --- | --- | --- |
| **Model 1** **UPFs consumption lower & middle tertile combined and depressive symptoms joint association** | | | | |
| LUND | 318 | Reference | Reference | Reference |
| LUD | 56 | 1.27 (0.62 – 2.60) | 1.30 (0.63 – 2.67) | 1.21 (0.58 – 2.53) |
| HUND | 158 | 1.30 (0.81 – 2.07) | 1.39 (0.86 – 2.25) | 1.37 (0.83 – 2.27) |
| HUD | 29 | 2.26 (1.10 – 4.64) | 2.65 (1.28 – 5.49) | 2.42 (1.05 – 5.55) |
| **Model 2 UPFs consumption lower & middle tertile combined and depressive symptoms/Antidepressant use joint association** | | | | |
| LUNDA | 295 | Reference | Reference | Reference |
| LUDA | 79 | 1.25 (0.65 – 2.38) | 1.25 (0.65 – 2.40) | 1.22 (0.63 – 2.36) |
| HUNDA | 147 | 1.14 (0.69 – 1.89) | 1.21 (0.73 – 2.03) | 1.21 (0.70 – 2.06) |
| HUDA | 40 | 2.72 (1.48 – 5.01) | 3.14 (1.69 – 5.82) | 3.00 (1.53 – 5.91) |
| LUND, lower/middle tertile of ultra-processed foods consumption and low depressive symptoms; LUD, lower/middle tertile of ultra-processed foods consumption and high depressive symptoms; HUND, higher tertile of ultra-processed foods consumption and low depressive symptoms; HUD, higher tertile of ultra-processed foods consumption and high depressive symptoms; LUNDA, lower and middle tertile of of ultra-processed foods consumption and low depressive symptoms and no antidepressant use; LUDA, lower and middle tertile of ultra-processed foods consumption and high depressive symptoms or antidepressant use; HUNDA, higher tertile of ultra-processed foods consumption and low depressive symptoms and no antidepressant; HUDA, higher tertile of ultra-processed foods consumption and high depressive symptoms or antidepressant.  *Fully adjusted model is adjusted for the following variables: age, sex, household income, education, ethnicity, born in Canada, smoking status, physical activity, daily alcohol consumption and BMI. | | | | |
